# Mitochondrial dynamics and colorectal cancer biology: mechanisms and potential targets

**DOI:** 10.1186/s12964-024-01490-4

**Published:** 2024-02-01

**Authors:** Zihong Wu, Chong Xiao, Jing Long, Wenbo Huang, Fengming You, Xueke Li

**Affiliations:** 1 Hospital of Chengdu University of Traditional Chinese Medicine, Chengdu, 610072 China; 2 Oncology Teaching and Research Department of Chengdu, University of Traditional Chinese Medicine, Chengdu, 610072 China; 3 Institute of Oncology, Chengdu University of Traditional Chinese Medicine, Chengdu, 610072 China

**Keywords:** Colorectal cancer, Mitochondrial dynamics, Fusion–fission, Drug targets

## Abstract

Colorectal cancer (CRC) is a significant public health concern, and its development is associated with mitochondrial dysfunction. Mitochondria can adapt to the high metabolic demands of cancer cells owing to their plasticity and dynamic nature. The fusion–fission dynamics of mitochondria play a crucial role in signal transduction and metabolic functions of CRC cells. Enhanced mitochondrial fission promotes the metabolic reprogramming of CRC cells, leading to cell proliferation, metastasis, and chemoresistance. Excessive fission can also trigger mitochondria-mediated apoptosis. In contrast, excessive mitochondrial fusion leads to adenosine triphosphate (ATP) overproduction and abnormal tumor proliferation, whereas moderate fusion protects intestinal epithelial cells from oxidative stress-induced mitochondrial damage, thus preventing colitis-associated cancer (CAC). Therefore, an imbalance in mitochondrial dynamics can either promote or inhibit CRC progression. This review provides an overview of the mechanism underlying mitochondrial fusion–fission dynamics and their impact on CRC biology. This revealed the dual role of mitochondrial fusion–fission dynamics in CRC development and identified potential drug targets. Additionally, this study partially explored mitochondrial dynamics in immune and vascular endothelial cells in the tumor microenvironment, suggesting promising prospects for targeting key fusion/fission effector proteins against CRC.

**Graphical Abstract:**

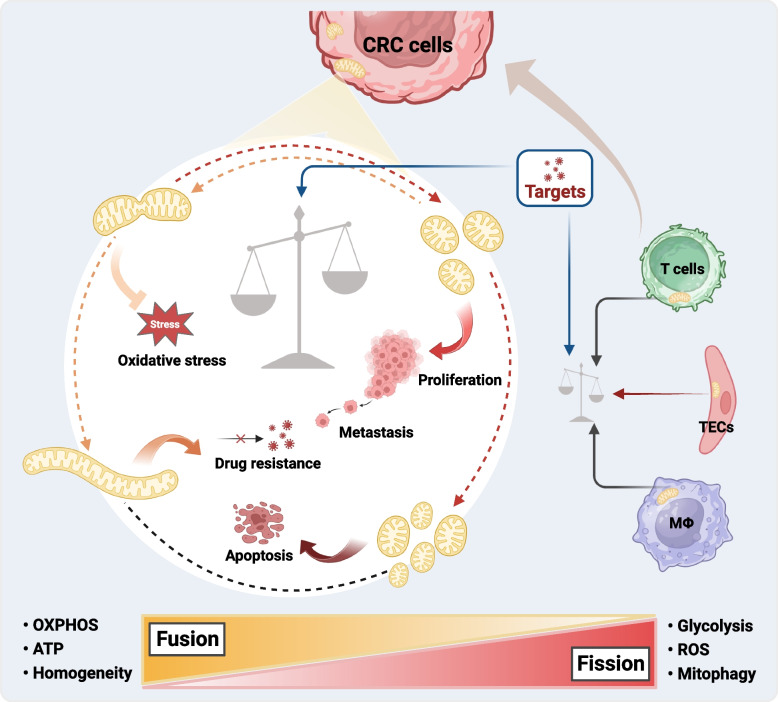

**Supplementary Information:**

The online version contains supplementary material available at 10.1186/s12964-024-01490-4.

## Introduction

Colorectal cancer (CRC) is the third most common cancer and second leading cause of cancer-related deaths in the United States [1]. Recent cancer statistics indicate that there will be an estimated 153,020 new CRC cases by 2023, resulting in 52,550 deaths [1, 2]. Although the overall incidence and mortality rates have decreased, there is a growing concern that the burden of CRC is shifting towards younger adults [3]. In recent years, significant advancements were made in CRC treatment through chemotherapy, molecular targeted therapies, and immune checkpoint inhibitors. However, recurrence and drug resistance hinder successful cancer treatment, resulting in a relatively poor prognosis, with a 5-year survival rate of approximately 60% [4, 5]. Moreover, approximately 20% of patients with CRC present metastases at the time of diagnosis, whereas 25% of patients with early-stage CRC develop metastases during follow-up [6]. Unfortunately, the prognosis of patients with metastatic CRC is worse, with a 5-year survival rate of below 20% [6]. Therefore, it is crucial to delve deeper into the key molecular events involved in colorectal carcinogenesis and progression and to explore new therapeutic targets.

Currently, metabolic reprogramming is the focus of oncological research. Recent evidence suggests that the unique metabolism of tumor cells characterized by reduced oxidative phosphorylation (OXPHOS) and increased glycolysis is regulated by mitochondrial dynamics [7–9]. Mitochondria are known as the ‘energy factories’ of eukaryotic cells that exhibit dynamic properties such as fusion, fission, and degradation, which are crucial for their optimal function in energy production [10, 11]. They play significant roles in various cellular processes, including cell differentiation, apoptosis, calcium homeostasis, innate immunity, and metabolism of fatty acids (FAs) and amino acids [12, 13]. These active organelles are transported along the cytoskeleton and can adopt different shapes, such as merging into long or interconnected tubules or splitting into small spheroids. These morphological changes are regulated by the opposing fusion and fission processes [14]. Continual fusion and fission events of mitochondrial membranes help regulate the morphology and quantity of mitochondria, ensuring their homogeneity and efficient functioning [14, 15]. Furthermore, imbalanced mitochondrial fusion–fission during the cell cycle appears to facilitate the entry of cancer cells into mitosis, thereby ensuring a proliferative and survival advantage [16].

‘Mitochondrial dynamics’ refers to various processes, such as fusion, fission, mitophagy, cristae remodeling, and transport. Mitophagy selectively eliminates dysfunctional mitochondria to regulate their quality and quantity [17]. Although mitophagy is influenced by mitochondrial dynamics and plays a significant role in CRC biology, these aspects were extensively discussed elsewhere [13, 18] and are beyond the scope of this review. This review focuses on the impact of fusion–fission dynamics on CRC cell biology. Here, we provide a brief overview of the mechanisms of mitochondrial fusion and fission, followed by a summary of how an imbalance in mitochondrial dynamics can facilitate or inhibit CRC development. Furthermore, this study highlights the potential of targeting key effector proteins involved in mitochondrial dynamics for CRC treatment.

## Overview of mitochondrial fusion–fission dynamics

Maintaining a balance between fusion and fission is crucial for preserving mitochondrial morphology and facilitating efficient exchange of contents between mitochondria.

**Fig. 1:**
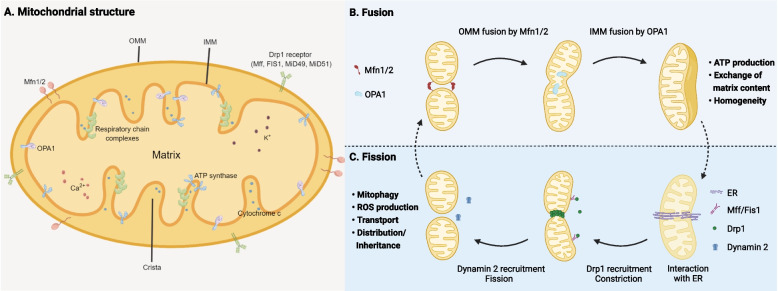
Mitochondrial structure and fusion–fission dynamics. **A** Mitochondrial structure, the mitochondrial lumen (matrix) is surrounded by the OMM and IMM. **B** Mitochondrial fusion involves outer membrane fusion and inner membrane fusion, facilitating ATP production and exchange of matrix content. **C** Mitochondrial fission involves ER interactions, Drp1 recruitment and constriction, and auxiliary segmentation of Dynamin 2, promoting ROS production, clearance of damaged mitochondria by autophagy, and equitable inheritance. ATP, adenosine triphosphate; Drp1, dynamin-related protein 1; ER, endoplasmic reticulum; FIS1, mitochondrial fission protein 1; IMM, inner mitochondrial membrane; Mff, mitochondrial fission factor; Mfn, mitofusins; OMM, outer mitochondrial membrane; OPA1, optic atrophy 1; ROS, reactive oxygen species

Mitochondrial fusion is defined as the complete fusion of two mitochondria through end-to-end collision [13]. Mitochondria are composed of two membranes: the outer mitochondrial membrane (OMM) and inner mitochondrial membrane (IMM). Fusion begins with outer membrane fusion, followed by inner membrane fusion, which occurs in close proximity. The IMM contains the lumen (matrix) of the mitochondria, an inner bordering membrane parallel to the OMM, and a deep convoluted polymorphic invagination known as the crista. The crista enlarges the surface area of the inner membrane and conceals components necessary for mitochondrial respiration. The OMM acts as a permeable platform that facilitates the convergence of cellular signals, which can be decoded and transmitted to mitochondria. It also establishes membrane contact with other organelles, such as the endoplasmic reticulum (ER), lysosomes, and melanosomes [14]. When the four lipid bilayers merge, the contents mix, and the matrix components diffuse to form a single, fused mitochondrion [16] (Fig. 1A). In addition to complete fusion, there is the so-called ‘kiss-and-run’ pattern of transient fusion. Unlike complete fusion, transient fusion occurs when two mitochondria come together, partially exchange intact membrane proteins, and separate, thereby maintaining their original topology. Transient fusion can occur even when optic atrophy 1 (OPA1) levels are too low or too high to support complete fusion. This type of fusion enhances the functional stability and plasticity of mitochondria and is necessary to support mitochondrial metabolism [19, 20].

Mitochondrial fusion is facilitated by three large GTPases from the dynamin superfamily: mitofusin 1(Mfn1), Mfn2, and OPA1. Mfn1 and Mfn2 are located on the outer membrane, and fusion begins with the docking of two trans-Mfn molecules to form homooligomeric and heterooligomeric complexes. Conformational changes in these complexes and the subsequent formation of oligomers contribute to the outer membrane fusion. Outer membrane fusion requires both Mfn1 and Mfn2 to be present; in the absence of either Mfn1 or Mfn2, the outer membrane fails to fuse [21]. OPA1 is involved in inner membrane fusion. Unlike Mfn1/2, the presence of OPA1 in only one of the two opposing mitochondria is sufficient to initiate inner membrane fusion [22] (Fig. 1B). Without OPA1, the cells only fuse the outer mitochondrial membrane fusion and fail to progress to inner membrane fusion. The intermediate products of failed fusion are eventually degraded by division, leading to mitochondrial fragmentation [23]. In addition, OPA1 plays a crucial role in maintaining the structure of the crista membrane, which is the primary site of OXPHOS. When OPA1 is deficient, the ultrastructure of the cristae is severely damaged, and respiratory chain complexes are significantly decreased [14].

Mitochondrial fission is the process of dividing a mitochondrion into two smaller mitochondria. This process begins with an interaction between the mitochondria and ER, causing mitochondrial tubule contraction [24]. The key step in fission is the recruitment and oligomerization of dynamin-related protein 1 (Drp1), a major mitochondrial fission protein whose activity is regulated by phosphorylation [25]. Recruitment of Drp1 to the mitochondria is dependent on several key receptors present on the outer membrane. One such receptor is mitochondrial fission factor (Mff), whose depletion causes substantial mitochondrial elongation and reduced Drp1 recruitment [26]. Other proteins, such as mitochondrial fission protein 1 (Fis1) and mitochondrial dynamics proteins of 49 and 51 kDa (MiD49 and MiD51), also play crucial roles in the recruitment process [16]. After being recruited to the mitochondria-ER contact site, Drp1 forms a helical structure in collaboration with actin, which initiates GTP hydrolysis and mediates the fission of the mitochondrial double membrane [27, 28]. Owing to certain biophysical limitations of Drp1, the final step of fission requires dynamin 2- and Golgi-derived PI (4)P-containing vesicles to induce membrane division to complete fission [29, 30] (Fig. 1C).

Although the mechanisms underlying fusion and fission are distinct, they work in tandem to ensure efficient transport, fair mitochondrial inheritance, and effective OXPHOS. A significant defect in either process can lead to mitochondrial dysfunction and potentially cancer development. With a better grasp of the basics of mitochondrial fusion and fission, we can examine the role of mitochondrial dynamics in CRC progression.

## Mitochondrial fission and CRC

**Fig. 2:**
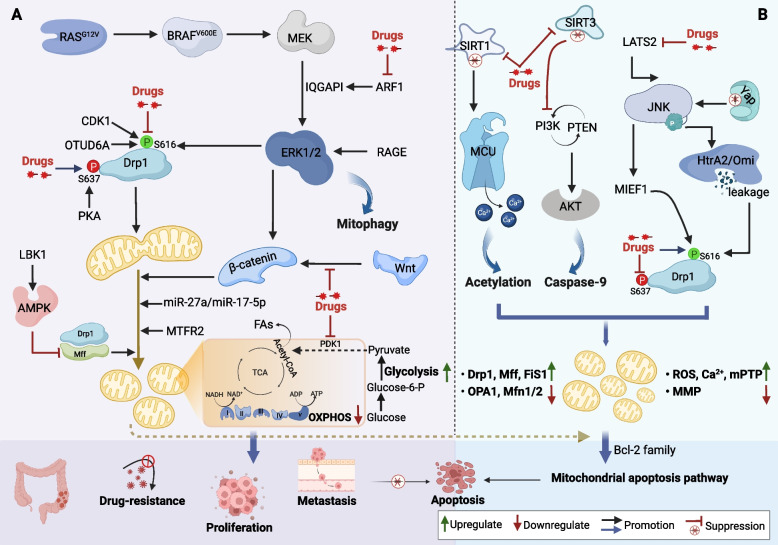
Mitochondrial fission and CRC biology: mechanisms and potential targets.** A** Fission promotes cell proliferation, metastasis, and drug resistance. **B** Excessive fission triggers mitochondria-mediated apoptosis. AMPK, AMP-activated protein kinase; CDK1, cyclin-dependent kinase 1; CRC, colorectal cancer; Drp1, dynamin-related protein 1; ERK, extracellular signal-regulated kinase; FIS1, mitochondrial fission protein 1; FAs, fatty acids; JNK, Jun N-terminal kinase; LATS2, large tumor suppressor kinase 2; LKB1, Liver kinase B1; MCU, mitochondrial calcium uniporter; MEK, mitogen-extracellular activated protein kinase; Mff, mitochondrial fission factor; MIEF, mitochondrial elongation factor 1; MMP, mitochondrial membrane potential; mPTP, mitochondrial permeability transition pore; MTFR2, mitochondrial fission regulator 2; OPA1, optic atrophy 1; OXPHOS, oxidative phosphorylation; PDK1, pyruvate dehydrogenase kinase 1; PTEN, phosphatase and tensin homolog; RAGE, receptor advanced glycation end product; ROS, reactive oxygen species; SIRT, Sirtuin; TCA, tricarboxylic acid cycle

In CRC cells, enhanced mitochondrial fission is a common phenomenon that promotes or inhibits CRC progression. To better understand this relationship, we will explore the molecules associated with fission and drugs that have been developed thus far (Fig. 2 and Supplementary Table 1).

### Fission promotes cell proliferation and migration

#### Key effector proteins/molecules mediating fission

Reducing the expression of the mitochondrial fission factor Drp1 results in elongated mitochondria, lowered mitochondrial membrane potential (MMP), increased cytochrome C release, and increased apoptosis in HCT116 and SW480 cells [31]. Drp1 also plays a crucial role in regulating FAs metabolism and mitochondrial morphology in CRC cells [32]. Treatment with FAs resulted in shorter average mitochondrial length and an increased number of mitochondria in HCT116 cells, indicating that FAs promote mitochondrial division. This involves Drp1 phosphorylation at Ser616 and enhanced interactions between Drp1 and Mff. However, this treatment did not affect the mRNA or protein levels of total Drp1 or other mitochondrial dynamics-related proteins, such as Mfn1, Mfn12, and OPA1. Functionally, activation of Drp1^S616^ promotes FAs oxidation, enhances Wnt/β-catenin pathway, and induces metabolic reprogramming [32]. In addition to FAs metabolism, mitochondrial fission reprograms glucose metabolism and promotes CRC cell growth, invasion, and migration. Activation of mitogen-activated protein kinase/extracellular signal-regulated kinase (MEK/ERK) signaling in CRC cells carrying *BRAF*^V600E^ promotes the phosphorylation of Drp1^S616^. However, there was no significant change in the level of Mfn1/2, resulting in more fragmented mitochondria and a glycolytic phenotype in tumor cells. This glycolytic phenotype is characterized by a significant increase in glucose uptake and lactate production. Furthermore, mitochondrial pyruvate dehydrogenase kinase 1 (PDK1) is a potential therapeutic target owing to its positive effects on mitochondrial fragmentation [33]. In HT-29 cell lines, the regulation of *Ras*^G12V^-induced cellular transformation is dependent on mitochondrial fission and Drp1, and the Drp1^S616^ phosphorylation status is sufficient to characterize transformation-induced mitochondrial dysfunction that can identify *BRAF*^V600E^-positive lesions [34]. ADP-ribosylation factor 1(ARF1) expression is linked to poor survival in CRC and activates ERK signaling by enhancing the interaction of IQ-domain GTPase-activating protein 1 (IQGAP1) with ERK and MEK, promoting mitochondrial division and colon tumorigenesis [35]. These findings suggest that targeting mitochondrial fission may aid the development of inhibitors of the oncogenic mitogen-activated protein kinase (MAPK) pathway [33–35].

Mitochondrial fission regulator 2 (MTFR2) is known for its role in regulating mitochondrial fission and is expressed at abnormal levels in various cancer tissues, including CRC tissues. In fact, a significant decrease in cancer cell proliferation, invasion, and migration was observed when MTFR2 was knocked down in HCT116 cells [36]. The miR-27a/Forkhead Box J3 (*FOXJ3)* axis regulates mitochondrial dynamics and biogenesis, resulting in shorter, fewer, and punctate mitochondria. Confocal microscopy showed an increase in mitochondrial abundance, accompanied by increased levels of mitochondrial electron transport chain (ETC) complexes and mitochondrial fusion proteins (Mfn1, Mfn2, and OPA1), and lower levels of fission proteins (Mff and Drp1) in CRC cells [37]. The ovarian tumor-associated protease deubiquitinase 6 A (*OTUD6A*) is overexpressed in human CRC tissues and stabilizes Drp1 through deubiquitination. The MDVD domain of Drp1 is the key region for its interaction with *OTUD6A*, and aberrant expression of *OTUD6A* in HCT116 and DLD1 cells prolongs the half-life of Drp1, thereby promoting cell proliferation and colony formation. Conversely, *OTUD6A* deletion leads to a significant increase in mitochondrial length and decrease in mitochondrial fragmentation [38]. Chemoresistance is a major barrier for effective cancer treatment. 5-Fluorouracil (5-FU)-, doxorubicin-, or oxaliplatin-resistant colon cancer cells show remarkable mitochondrial fragmentation owing to reduced fusion and increased division, which is consistent with low ROS accumulation [39–41]. Mechanistically, mettl14-dependent maturation of pri-miR-17 directly induces mitochondrial fission, leading to 5-FU chemoresistance [40]. Although Drp1 phosphorylation (Ser616) may be involved in chemoresistance, high Drp1 phosphorylation triggered by ERK can activate autophagy through the receptor advanced glycation end product (RAGE)/ERK1/2 pathway, resulting in chemoresistance and regeneration of cancer cells [41].

#### Compounds inhibiting mitochondrial fission

In addition to the above research on key effector proteins or molecules, multiple studies on the anti-cancer activity of naturally occurring compounds or specific drugs confirmed that mitochondrial fission promotes CRC progression. Mitochondrial division inhibitor 1 (Mdivi-1) is a commonly used Drp1 inhibitor that inhibits mitochondrial fission, reduces oxidative metabolism in CRC cells, and impedes cell proliferation [42]. HCT116 cells treated with ellagic acid (a polyphenolic compound) resulted in hyperfusion in the originally small and punctate mitochondria, which leads to reduced mitochondrial membrane potential and impaired mitochondrial respiration. Additionally, dephosphorylation of Drp1^S616^ was observed, along with decreased expression of cell proliferation and cell cycle markers, cyclin-dependent kinase 1 (CDK1), cyclin B, and Ki67 [43]. This phenomenon also occurs in CRC cells exposed to Paris Saponin II [44], sodium butyrate [45], and the Wnt/β-catenin signaling inhibitor ICG-001 [46]. Paris saponin II dephosphorylates Drp1^S616^ and inhibits Drp1 recruitment by activating the nuclear factor-kappa B (NF-κB) pathway, triggering cell cycle arrest and apoptosis [44]. Furthermore, ICG-001 activates the early ER stress response to exert anti-proliferative activity [46]. Corosolic acid is a natural pentacyclic triterpenoid that suppresses Drp1 phosphorylation at Ser616 (CDK1-dependent) and induces Drp1 phosphorylation at Ser637 (protein kinase-dependent). Heterodimerization of human epidermal growth factor receptor 2 (HER2) and HER3 inhibits the migration of Drp1 to the mitochondrial surface, thereby inhibiting mitochondrial division. Although corosolic acid did not significantly affect OPA1 and Mfn1/2 expression, it shows promise as a novel inhibitor of HER2/HER3 heterodimerization in CRC [47]. Atractylenolide I increases ROS content, impairs mitochondrial membrane integrity, and induces apoptosis in CRC cells. It also suppresses NLRP3 (NACHT, LRR, and PYD domain–containing protein 3) inflammasome activation in colitis-associated cancer (CAC) by inhibiting Drp1-mediated mitochondrial fission [48]. Pectin is a polysaccharide that decreases Drp-1 expression and increases the expression of proteins linked to mitochondrial fusion and cellular senescence, such as β-galactosidase and p53, resulting in reduced cell viability and cellular senescence [49]. The anti-allergic drug azelastine may exert anti-colon cancer effects by inhibiting ARF1-mediated mitochondrial fission via the ERK signaling pathway [35]. Metformin is a drug commonly used to treat type 2 diabetes mellitus that delays the progression of CAC by activating the liver kinase B1/AMP-activated protein kinase (*LKB1*/AMPK) pathway and protects the mitochondrial structure of colonic epithelial cells while inhibiting H_2_O_2_-induced mitochondrial division [50].

Taken together, mitochondrial fission is commonly enhanced in CRC owing to aberrantly expressed genes. This process occurs through ERK-mediated phosphorylation of Drp1 at Ser616 while inhibiting phosphorylation at Ser637, leading to metabolic reprogramming, enhanced cell proliferation, invasion, migration, chemoresistance, and inhibition of apoptosis. Mitochondrial fission primarily activates the oncogenic MAPK pathway. The main focus of some anti-cancer drugs is to inhibit mitochondrial division by preventing the recruitment of Drp1, inhibiting ERK1/2 phosphorylation, and dephosphorylating Drp1 at Ser616 (Fig. 2A). These findings will aid the development of new small-molecule inhibitors of CRC.

### Excessive fission induces apoptosis

#### Molecules mediating excessive fission

Normal mitochondrial fission plays a crucial role in enhancing the efficiency of energy metabolism. However, excessive fission adversely affects the viability of CRC cells. Mitochondrial deacetylases, Sirtuin-3 (SIRT3), and SIRT1 are enzymes found within the mitochondria that actively promote the proliferation and migration of tumor cells. Marked mitochondrial fragmentation is observed when SIRT3 and SIRT1 are deficient or inhibited in CRC cells, accompanied by ROS overproduction, decreased MMP, open mitochondrial permeability transition pore (mPTP), upregulated fission indicators, and decreased fusion indicators, ultimately inducing abnormal mitochondrial division and CRC apoptosis [51, 52]. SIRT3 deletion activates caspase-9-related death programs by inhibiting Akt/phosphatase and tensin homolog (PTEN) signaling [51], whereas SIRT1 inhibition primarily increases the acetylation of the mitochondrial calcium uniporter (MCU) at the K332 site, resulting in mitochondrial Ca^2+^ enrichment and depolarization, and the SIRT1 inhibitor Inauhzin targets this process [52]. Decreased expression of large tumor suppressor kinase 2 (LATS2) may cause cancer cells to become resistant to 5-FU chemotherapy. Conversely, LATS2 upregulation in SW480 cells induces higher levels of mitochondrial division, activates the Bax-related mitochondrial apoptotic pathway, decreases ATP content, and enhances apoptotic response to 5-FU [53]. Mechanistically, LATS2 overexpression further amplifies Jun N-terminal kinase (JNK)-mitochondrial elongation factor 1 (MIEF1)-related mitochondrial fission activated by 5-FU [53]. Matrine is a natural compound that activates MIEF1–related mitochondrial fission and inhibits the survival of SW480 cells through the LATS2-Hippo pathway [54]. YAP overexpression was observed in human rectal cancer cells. YAP deficiency contributes to the activation of JNK/Drp1^S616^ and the recruitment of Drp1, which causes mitochondrial fission. These abnormal mitochondrial divisions result in the leakage of HtrA2/Omi into the cytoplasm, which ultimately induces apoptosis in rectal cancer cells via the mitochondrial apoptosis pathway [55].

Although several studies have reported increased Drp1 expression in CRC, its exact role in CRC development remains unclear and requires further investigation. To investigate the involvement of Drp1 in colon cancer, Kim et al. [56] analyzed the levels of Drp1 in human colon cancer tissues; Drp1 levels were lower and higher than those in adjacent normal tissues in 75% (9 pairs) and 25% (3 pairs) of the tumor tissues, respectively. The study also revealed a significant decrease in Drp1 levels, specifically in mid- to late-stage colon cancer (*P* < 0.01), suggesting a potential association between the lack of Drp1 or a deficiency in the mitochondrial division and progression of colon cancer. Furthermore, the decline in Drp1 levels was greater in male patients than in female patients. These findings are in contrast with those of previous studies and indicate that the effects of changes in Drp1 levels may vary depending on the cell type, tissue, or physiological context.

#### Compounds enhancing mitochondrial fission

Several compounds, including inauhzin [52], matrine [54], Tanshinone IIA [57, 58], lycorine [59], and *Aloe gel* glucomannan [60] were developed to induce mitochondrial fission in CRC cells. Tanshinone IIA activates JNK-Mff and Mst1-Hippo signaling pathways, resulting in apoptosis and inhibition of cell proliferation [57, 58]. Lycorine and *Aloe gel* glucomannan increase mitochondrial fission and mitophagy signaling in colon cancer cells, leading to ROS overproduction, which contributes to cell death [59, 60]. Mitochondrial fission also plays an important role in chemotherapy-induced apoptosis. Treatment with triptolide and an apoptosis inducer kit (AIK) in HCT-116 cells upregulates the levels of p-Drp1^S616^ and induces mitochondrial fission. Similarly, camptothecin treatment increases the p-Drp1^S616^/ p-Drp1^S637^ ratio in the DLD1 cell line [61]. Myoferlin (MYOF) is a vital vesicular transporter protein, and its small-molecule inhibitor (YQ456) can dephosphorylate Drp1 at S637 in CRC cells, resulting in sustained mitochondrial fission and, ultimately, cell death [62]. These findings suggest that the activation of mitochondrial fission may contribute to halting CRC progression. However, further studies are required to fully elucidate these processes.

In summary, mitochondrial fission appears to play a dual role in CRC progression. Activated Drp1^S616^ promotes mitochondrial fission, metabolic reprogramming, activation of oncogenic signaling pathways, cell proliferation, invasion, migration, and resistance to chemotherapy. However, excessive mitochondrial fission owing to deficiency or overexpression of key genes triggers the Bcl-2 family-mediated mitochondrial apoptotic pathway (Fig. 2B). Therefore, while mitochondrial fission enhances CRC cell growth, excessive fission can induce apoptosis. This evidence could be valuable to identify targeted inhibitors for CRC.

## Mitochondrial fusion and CRC

**Fig. 3:**
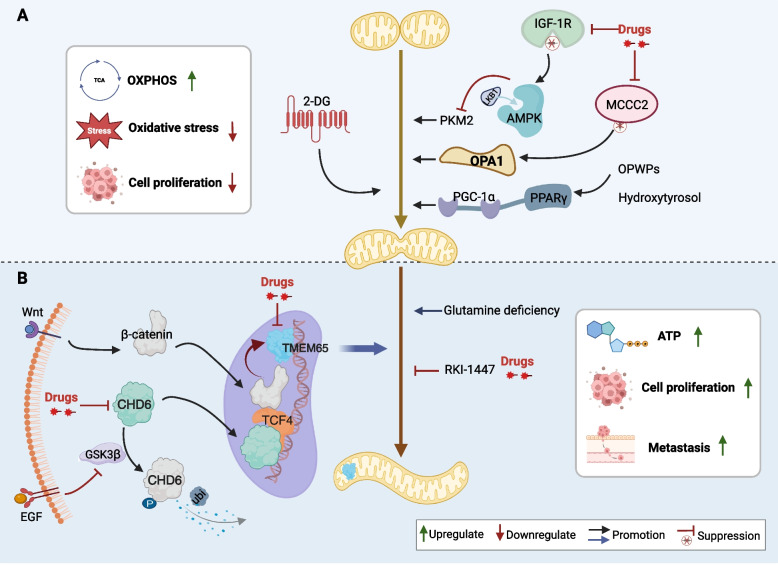
Mitochondrial fusion and CRC biology: mechanisms and potential targets.** A** Fusion attenuates oxidative stress-induced mitochondrial damage and inhibits the development of CAC. **B** Excessive fusion promotes cell proliferation, metastasis, and apoptosis resistance. 2-DG, 2-deoxy-D-glucose; ATP, adenosine triphosphate; CAC, colitis-associated cancer; CHD6, chromodomain helicase 6; CRC, colorectal cancer; GSK3β, glycogen synthase kinase-3β; OPA1, optic atrophy 1; OPWPs, oil production waste products; OXPHOS, oxidative phosphorylation; TCA, tricarboxylic acid cycle; TMEM65, transmembrane protein 65

Mitochondrial fusion is the opposite process of mitochondrial fission, and its dysfunction plays an important role in the progression of CRC (Fig. 3 and Supplementary Table 1).

### Fusion attenuates oxidative stress-induced mitochondrial damage

Mitochondrial fusion may inhibit the development of CAC in the early stages. Insulin-like growth factor-1 receptor (IGF-1R) is considered a significant element in CRC owing to its potential to encourage cell cycle advancement and hinder apoptosis. Heterozygous knockdown of IGF-1R safeguards colonic epithelial cells from oxidative stress-induced mitochondrial damage through the LKB1/AMPK signaling pathway and activates fusion to maintain the structural integrity of mitochondria, which prevents colitis-associated cancers induced by azoxymethane/dextran sodium sulfate (AOM/DSS) [63]. Knockdown of the mitochondrial gene *MCCC2* inhibits CRC cell proliferation, invasion, and migration, which is mainly achieved by upregulating the expression of the fusion markers MFN and OPA1 [64]. A study on the mitochondrial biogenesis and kinetics of CRC cells exposed to oil production waste products (OPWPs) extracts and hydroxytyrosol shows that treated CRC cells showed elongated and tubular mitochondria, forming a complex network structure [65]. The levels of Mfn1/2 were increased, whereas those of Mff were significantly reduced. Mechanistically, OPWPs extracts and hydroxytyrosol promote mitochondrial fusion and upregulate the OXPHOS pathway via the PPARγ/PGC-1α axis, ultimately inhibiting cell proliferation and inducing apoptosis [65]. Tumor cells rely on glycolysis for ATP production. Intervention of colon cancer cells with 2-deoxy-D-glucose (2-DG) causes an imbalance in mitochondrial fusion and fission proteins, which mainly upregulates Mfn1/2 and downregulates Drp1. This results in mitochondrial fusion and network structure formation, accompanied by a notable inhibition of glycolysis [33, 66]. Interestingly, this intervention had no effect on the viability of colon cancer cells [66] (Fig. 3A).

### Excessive fusion promotes cell proliferation and migration

The chromodomain helicase DNA-binding protein (CHD) family has been linked to tumors. In particular, CDH6 is overexpressed in CRC cells. Aberrant activation of the EGF signal impedes glycogen synthase kinase-3β (GSK3β)-mediated CHD6 ubiquitination and degradation, resulting in structurally stable CHD6 entering the nucleus and binding to *transcription factor 4 (TCF4)* and β-catenin in response to Wnt signaling. This enhances the transcription of transmembrane protein 65 (TMEM65), a mitochondrial inner membrane protein, leading to sustained mitochondrial fusion, ATP overproduction, tumor growth, and metastasis. Knocking out the CHD6 gene in CRC cells shortens mitochondrial length and reduces the number of cristae, ultimately inducing apoptosis [67]. The absence of glutamine in cancer cells can cause a significant increase in ROS levels, leading to structural damage to mitochondria. In response, mitochondria fuse to dilute damaged proteins and repair damage, thereby maintaining the integrity of mitochondrial DNA [68]. Activation of mitochondrial depolarization in CRC cells was observed upon using a new Rho/ROCK inhibitor, RKI-1447. This activation leads to cleavage of OPA1 protein, resulting in impaired mitochondrial fusion, a decrease in mitochondrial respiration rate, excessive production of ROS, an increase in the expression of ER stress-associated molecules like p-eIF2α and CHOP, and ultimately induces ER stress-associated cell apoptosis [69] (Fig. 3B).

In summary, excessive mitochondrial fusion promotes cancer cell growth by generating large amounts of energy and repairing damaged mitochondrial structures. However, mitochondrial fusion can also protect the intestinal epithelial cells from oxidative stress-induced damage, thereby preventing the development of CAC. Additionally, mitochondrial fusion can inhibit the glycolytic pathway, which may slow CRC progression. However, further studies are required to fully understand these findings.

## Mitochondrial dynamics in the tumor microenvironment

**Fig. 4:**
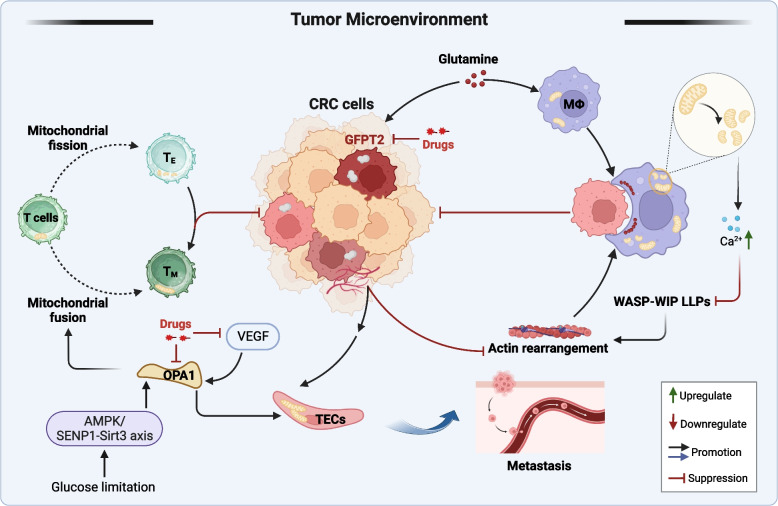
Mitochondrial dynamics in the tumor microenvironment. Mitochondrial fusion–fission dynamics in macrophages and T cells correlate with their immune activity. Targeting mitochondrial dynamics in immune cells enhances antitumor immunity. Mitochondrial fusion in TECs promotes angiogenesis and hematogenous metastasis. CRC, colorectal cancer; LLPs, liquid-liquid phase separations; OPA1, optic atrophy 1; TECs, tumor endothelial cells; VEGF, vascular endothelial growth factor; WASP–WIP, Wiskott–Aldrich syndrome protein–Wiskott–Aldrich syndrome interacting protein

Imbalances in mitochondrial dynamics were observed in CRC cells and in immune and endothelial cells in the tumor microenvironment. Recent studies demonstrate the critical role of mitochondrial division in macrophages for the effective phagocytosis of live tumors and apoptotic cells [70–72]. Furthermore, mitochondrial fission is essential to initiate the immune response in bone marrow-derived macrophages, enabling them to regulate tumor metastasis [73]. The phagocytic effect of macrophages is enhanced when they are stimulated with therapeutic monoclonal antibodies [70]. Mechanistically, mitochondrial fission increases the intracytoplasmic calcium ion concentration, which subsequently disrupts the liquid-liquid phase separation (LLP) of the Wiskott-Aldrich syndrome protein–Wiskott-Aldrich syndrome interacting protein (WASP–WIP) complex in macrophages and promotes actin rearrangement, ultimately enhancing the phagocytic activity of macrophages. Overexpression of a protease competing with macrophages for glutamine in colon cancer cells [glutamine-fructose-6-phosphate transaminase 2 (GFPT2)] can impair mitochondrial fission in macrophages, leading to a weakened phagocytic response and drug resistance [70]. The results of this study indicated that focusing on the process of mitochondrial fission in macrophages could potentially enhance the effectiveness of therapeutic antibodies. The differentiation of T cells into effector T (T_E_) cells (which divide mitochondria) or memory T (T_M_) cells (which fuse mitochondria) is closely associated with mitochondrial dynamic-mediated metabolic reprogramming [74]. He et al. [75] observed a notable tumor growth delay in Sirt3 K223R-OT1 T_M_  mouse model upon injection of OVA-expressing mouse MC38 (MC38-OVA) tumor cells. Mechanistically, activation of the SENP1-Sirt3 axis could inhibit OPA1 fragmentation in T_M_ cells and enhance mitochondrial fusion, thereby promoting T cell memory development.These findings suggest that activating mitochondrial fission in macrophages or mitochondrial fusion in T cells may improve antitumor immunity (Fig. 4).

In addition to immune cells, the mitochondrial dynamics of tumor endothelial cells (TECs) are closely linked to cancer cell proliferation and metastasis. Vascular endothelial growth factor (VEGF) stimulates the expression of the fusion protein OPA1 in endothelial cells, inhibits the NF-κB pathway, promotes angiogenesis and lymphangiogenesis, and induces CRC cell growth and metastasis [76] (Fig. 4). Cachexia that leads to muscle and fat depletion is a major complication of CRC. In C26 tumor-bearing mice, treatment with or without Folfiri (a chemotherapeutic agent) causes muscle atrophy and reduces the expression of mitochondrial fusion markers (OPA1 and Mfn2), fission markers (Drp1), and biogenesis markers (PGC-1α) in muscle cells. These findings suggest that tumors and chemotherapy can cause muscle loss through similar mechanisms, leading to changes in mitochondrial homeostasis. This highlights the potential of targeting myocyte mitochondrial dynamics to develop combination therapeutic strategies that can effectively counteract tumor growth and mitigate the side effects of chemotherapy [77].

## Conclusions and future perspectives

Recent studies have focused on discovering new preventive and therapeutic methods to combat CRC. Mitochondria play an important role in cellular metabolism. Cancer cells often reprogram their energy metabolism pathways to promote glycolysis and reduce the use of OXPHOS; this is known as the Warburg effect [78]. Mitochondrial function is closely associated with mitochondrial dynamics. Therefore, an in-depth understanding of mitochondrial dynamics is crucial to elucidating the pathological processes of CRC. This review reveals that mitochondrial fission/fusion plays a dual role in the development of CRC, with Drp1 and OPA1 serving as key effector proteins that mediate mitochondrial fission/fusion. Enhanced mitochondrial fission promotes the metabolic reprogramming of CRC cells, leading to cell proliferation, invasion, metastasis, and chemoresistance. In contrast, excessive fission activates the mitochondrial apoptotic pathway. Abnormal mitochondrial fusion results in ATP overproduction and abnormal tumor proliferation, whereas moderate fusion protects intestinal epithelial cells from oxidative stress-induced mitochondrial damage and prevents CAC. These findings indicate promising prospects for the development of inhibitors or promoters that target key effector proteins involved in mitochondrial dynamics in CRC cells. Such interventions can inhibit cell proliferation, migration, and drug resistance by restoring the fusion–fission balance or promoting apoptosis by inducing a fusion–fission imbalance. Furthermore, emerging evidence highlights the potential of interventions in the mitochondrial fusion–fission dynamics of immune cells and TECs in the intestinal tumor microenvironment to enhance immunotherapy.

A better understanding of the role of mitochondrial dynamics in CRC development, requires future studies to focus on several key areas. First, further investigation is needed to determine the structure-function relationships of key proteins involved in mitochondrial dynamics to identify potential drug targets. Second, it is important to clarify the causal relationship between imbalances in mitochondrial dynamics in CRC cells and various cellular processes. This includes determining whether it is the primary cause or a secondary effect of certain pathological processes and investigating whether the effects are consistent across different cell types. Third, current studies have oversimplified the relationship between mitochondrial elongation and fission and their respective effects on OXPHOS and glycolysis. Future research should explore the intricate relationship between mitochondrial morphology and energy metabolic pathways. In summary, mitochondrial fusion–fission dynamics are causally linked to various pathological processes in CRC cells, including energy metabolism, proliferation, invasion, migration, apoptosis, and drug resistance. Ongoing drug design efforts targeting the key effector proteins of mitochondrial fusion/fission have the potential to prevent and treat CRC.

### Supplementary Information

**Additional file 1: Supplementary Table 1.** The role of mitochondrial dynamics in CRC.

## Data Availability

Not applicable.

## Abbreviations

2-DG: 2-deoxy-D-glucose
AIK: Apoptosis inducer kit
AMPK: AMP-activated protein kinase
AOM/DSS: Azoxymethane/Dextran Sodium Sulfate
ARF1: ADP-ribosylation factor 1
ATP: Adenosine triphosphate
CAC: Colitis-associated cancer
CDK1: Cyclin dependent dinase 1
CDX: Cell derived xenograft
CHD: Chromodomain helicase DNA binding protein
CRC: Colorectal cancer
Drp1: Dynamin-related protein 1
ER: Endoplasmic reticulum
ERK: Extracellular signal-regulated kinase
ETC: Electron transport chain
FAs: Fatty acids
Fis1: Mitochondrial fission protein 1
FOXJ3: Forkhead Box J3
GFPT2: Glutamine-fructose-6-phosphate transaminase 2
GSK3β: Glycogen synthase kinase-3β
HER: Human epidermal growth factor receptor
IMM: Inner mitochondrial membrane
JNK: Jun N-terminal kinases
LATS2: Large tumor suppressor kinase 2
LKB1: Liver kinase B1
LLPs: Liquid-liquid phase separation
MAPK: Mitogen-activated protein kinases
MCU: Mitochondrial calcium uniporter
Mdivi-1: Mitochondrial-division inhibitor 1
MEK: Mitogen-extracellular activated protein kinase
Mff: Mitochondrial fission factor
Mfn: Mitofusins
MiD49 and MiD51: Mitochondrial dynamics proteins of 49 and 51 kDa
MIEF1: Mitochondrial elongation factor 1
MMP: Mitochondrial membrane potential
mPTP: Mitochondrial permeability transition pore
MTFR2: Mitochondrial fission regulator 2
MYOF: Myoferlin
NF-κB: Nuclear factor-kappa B
NLRP3: NACHT,LRR,and PYD domain–containing protein 3
OMM: Outer mitochondrial membrane
OPA1: Optic atrophy 1
OPWPs: Oil production waste products
OTUD6A: Ovarian tumor-associated protease deubiquitinase 6 A
OXPHOS: Oxidative phosphorylation
PDK1: Pyruvate dehydrogenase kinase 1
PDX: Patient-derived xenograft
PTEN: Phosphatase and tensin homologue
RAGE: Receptor advanced glycation end product
ROS: Reactive oxygen species
SIRT3: Sirtuin-3
TCA: Tricarboxylic acid cycle
TCF4: Transcription factor 4
TECs: Tumor endothelial cells
TMEM65: Transmembrane protein 65
VEGF: Vascular endothelial growth factor
IGF-1R: Insulin-like growth factor-1 receptor
IQGAP1: IQ-domain GTPase activating protein 1. WASP–WIP,Wiskott–Aldrich syndrome protein–Wiskott–Aldrich syndrome interacting protein
5-FU: 5-Fluorouracil
2-DG: 2-deoxy-D-glucose

## References

[1] Siegel RL, Wagle NS, Cercek A, Smith RA, Jemal A (2023). Colorectal cancer statistics, 2023. CA Cancer J Clin.

[2] Siegel RL, Miller KD, Wagle NS, Jemal A (2023). Cancer statistics, 2023. CA Cancer J Clin.

[3] Siegel RL, Jakubowski CD, Fedewa SA, Davis A, Azad NS (2020). Colorectal Cancer in the Young: Epidemiology, Prevention, Management. Am Soc Clin Oncol Educ Book.

[4] Luo M, Yang X, Chen H-N, Nice EC, Huang C. Drug resistance in colorectal cancer: an epigenetic overview. Biochim et Biophys Acta (BBA) - Reviews Cancer. 2021;1876(2). 10.1016/j.bbcan.2021.188623. 10.1016/j.bbcan.2021.188623 34481016

[5] Hossain MS, Karuniawati H, Jairoun AA, Urbi Z, Ooi DJ, John A, et al. Colorectal Cancer: a review of Carcinogenesis, Global Epidemiology, Current challenges, risk factors, preventive and treatment strategies. Cancers. 2022;14(7). 10.3390/cancers14071732. 10.3390/cancers14071732 PMC8996939 35406504

[6] Biller LH, Schrag D (2021). Diagnosis and treatment of metastatic colorectal Cancer: a review. Jama-Journal of the American Medical Association.

[7] Sessions DT, Kashatus DF (2021). Mitochondrial dynamics in cancer stem cells. Cell Mol Life Sci.

[8] Bonnay F, Veloso A, Steinmann V, Köcher T, Abdusselamoglu MD, Bajaj S (2020). Oxidative metabolism drives immortalization of neural stem cells during Tumorigenesis. Cell.

[9] Li T, Han JB, Jia LJ, Hu X, Chen LQ, Wang YG (2019). PKM2 coordinates glycolysis with mitochondrial fusion and oxidative phosphorylation. Protein Cell.

[10] Chen W, Zhao H, Li Y (2023). Mitochondrial dynamics in health and disease: mechanisms and potential targets. Signal Transduct Target Ther.

[11] Mishra P, Chan DC (2016). Metabolic regulation of mitochondrial dynamics. J Cell Biol.

[12] Zhou Z, Fan Y, Zong R, Tan K (2022). The mitochondrial unfolded protein response: a multitasking giant in the fight against human diseases. Ageing Res Rev.

[13] Chan DC (2020). Mitochondrial dynamics and its involvement in Disease. Annu Rev Pathol.

[14] Giacomello M, Pyakurel A, Glytsou C, Scorrano L (2020). The cell biology of mitochondrial membrane dynamics. Nat Rev Mol Cell Biol.

[15] Klos P, Dabravolski SA. The role of Mitochondria Dysfunction in Inflammatory Bowel diseases and Colorectal Cancer. Int J Mol Sci. 2021;22(21). 10.3390/ijms222111673. 10.3390/ijms222111673 PMC8584106 34769108

[16] Chen H, Chan DC (2017). Mitochondrial dynamics in regulating the unique phenotypes of Cancer and Stem cells. Cell Metab.

[17] Onishi M, Yamano K, Sato M, Matsuda N, Okamoto K (2021). Molecular mechanisms and physiological functions of mitophagy. EMBO J.

[18] Kasprzak A. Autophagy and the insulin-like growth factor (IGF) system in Colonic cells: implications for colorectal neoplasia. Int J Mol Sci. 2023;24(4). 10.3390/ijms24043665. 10.3390/ijms24043665 PMC9959216 36835075

[19] Liu X, Weaver D, Shirihai O, Hajnoczky G (2009). Mitochondrial ‘kiss-and-run’: interplay between mitochondrial motility and fusion–fission dynamics. EMBO J.

[20] Wang S, Xiao W, Shan S, Jiang C, Chen M, Zhang Y (2012). Multi-patterned dynamics of mitochondrial fission and fusion in a living cell. PLoS ONE.

[21] Zacharioudakis E, Agianian B, Mv VK, Biris N, Garner TP, Rabinovich-Nikitin I et al. Modulating mitofusins to control mitochondrial function and signaling. Nat Commun. 2022;13(1)3775. 10.1038/s41467-022-31324-1. 10.1038/s41467-022-31324-1 PMC9262907 35798717

[22] Gao S, Hu JJ (2021). Mitochondrial Fusion: the machineries in and out. Trends Cell Biol.

[23] Liu C, Han Y, Gu X, Li M, Du Y, Feng N (2021). Paeonol promotes Opa1-mediated mitochondrial fusion via activating the CK2alpha-Stat3 pathway in diabetic cardiomyopathy. Redox Biol.

[24] Zhou Z, Torres M, Sha H, Halbrook CJ, Van den Bergh F, Reinert RB (2020). Endoplasmic reticulum-associated degradation regulates mitochondrial dynamics in brown adipocytes. Science.

[25] Kalia R, Wang RY, Yusuf A, Thomas PV, Agard DA, Shaw JM (2018). Structural basis of mitochondrial receptor binding and constriction by DRP1. Nature.

[26] Passmore JB, Carmichael RE, Schrader TA, Godinho LF, Ferdinandusse S, Lismont C et al. Mitochondrial fission factor (MFF) is a critical regulator of peroxisome maturation. Biochim Biophys Acta Mol Cell Res. 2020;1867(7): 118709. 10.1016/j.bbamcr.2020.118709. 10.1016/j.bbamcr.2020.118709 PMC7262603 32224193

[27] Kleele T, Rey T, Winter J, Zaganelli S, Mahecic D, Lambert P (2021). Distinct fission signatures predict mitochondrial degradation or biogenesis. Nature.

[28] Jimah JR, Hinshaw JE (2019). Structural insights into the mechanism of Dynamin Superfamily proteins. Trends Cell Biol.

[29] Lee JE, Westrate LM, Wu HX, Page C, Voeltz GK (2016). Multiple dynamin family members collaborate to drive mitochondrial division. Nature.

[30] Nagashima S, Tabara LC, Tilokani L, Paupe V, Anand H, Pogson JH (2020). Golgi-derived PI(4)P-containing vesicles drive late steps of mitochondrial division. Science.

[31] Inoue-Yamauchi A, Oda H (2012). Depletion of mitochondrial fission factor DRP1 causes increased apoptosis in human colon cancer cells. Biochem Biophys Res Commun.

[32] Xiong X, Hasani S, Young LEA, Rivas DR, Skaggs AT, Martinez R (2022). Activation of Drp1 promotes fatty acids-induced metabolic reprograming to potentiate wnt signaling in colon cancer. Cell Death Differ.

[33] Padder RA, Bhat ZI, Ahmad Z, Singh N, Husain M (2020). DRP1 promotes BRAF(V600E)-Driven tumor progression and metabolic reprogramming in Colorectal Cancer. Front Oncol.

[34] Serasinghe MN, Wieder SY, Renault TT, Elkholi R, Asciolla JJ, Yao JL (2015). Mitochondrial division is requisite to RAS-induced transformation and targeted by oncogenic MAPK pathway inhibitors. Mol Cell.

[35] Hu HF, Xu WW, Li YJ, He Y, Zhang WX, Liao L (2021). Anti-allergic drug azelastine suppresses colon tumorigenesis by directly targeting ARF1 to inhibit IQGAP1-ERK-Drp1-mediated mitochondrial fission. Theranostics.

[36] Xie Y, Chen R, Yan L, Jia Z, Liang G, Wang Q. Transcription factor HOXC10 activates the expression of MTFR2 to regulate the proliferation, invasion and migration of colorectal cancer cells. Mol Med Rep. 2021;24(5). 10.3892/mmr.2021.12437. 10.3892/mmr.2021.12437 PMC8456344 34523692

[37] Barisciano G, Leo M, Muccillo L, Pranzini E, Parri M, Colantuoni V, et al. The miR-27a/FOXJ3 Axis Dysregulates mitochondrial homeostasis in Colorectal Cancer cells. Cancers (Basel). 2021;13(19). 10.3390/cancers13194994. 10.3390/cancers13194994 PMC8507763 34638478

[38] Shi L, Liu J, Peng Y, Zhang J, Dai X, Zhang S (2020). Deubiquitinase OTUD6A promotes proliferation of cancer cells via regulating Drp1 stability and mitochondrial fission. Mol Oncol.

[39] Locatelli L, Cazzaniga A, Fedele G, Zocchi M, Scrimieri R, Moscheni C (2021). A comparison of doxorubicin-resistant Colon cancer LoVo and leukemia HL60 cells: common features, different underlying mechanisms. Curr Issues Mol Biol.

[40] Sun K, Chen L, Li Y, Huang B, Yan Q, Wu C (2023). METTL14-dependent maturation of pri-miR-17 regulates mitochondrial homeostasis and induces chemoresistance in colorectal cancer. Cell Death Dis.

[41] Huang CY, Chiang SF, Chen WT, Ke TW, Chen TW, You YS (2018). HMGB1 promotes ERK-mediated mitochondrial Drp1 phosphorylation for chemoresistance through RAGE in colorectal cancer. Cell Death Dis.

[42] Dai W, Wang G, Chwa J, Oh ME, Abeywardana T, Yang Y (2020). Mitochondrial division inhibitor (mdivi-1) decreases oxidative metabolism in cancer. Br J Cancer.

[43] Yakobov S, Dhingra R, Margulets V, Dhingra A, Crandall M, Kirshenbaum LA (2023). Ellagic acid inhibits mitochondrial fission protein Drp-1 and cell proliferation in cancer. Mol Cell Biochem.

[44] Chen M, Ye K, Zhang B, Xin Q, Li P, Kong AN (2019). Paris Saponin II inhibits colorectal carcinogenesis by regulating mitochondrial fission and NF-kappaB pathway. Pharmacol Res.

[45] Tailor D, Hahm ER, Kale RK, Singh SV, Singh RP (2014). Sodium butyrate induces DRP1-mediated mitochondrial fusion and apoptosis in human colorectal cancer cells. Mitochondrion.

[46] Zinecker H, Ouaret D, Ebner D, Gaidt MM, Taylor S, Aulicino A (2017). ICG-001 affects DRP1 activity and ER stress correlative with its anti-proliferative effect. Oncotarget.

[47] Zhang BY, Zhang L, Chen YM, Qiao X, Zhao SL, Li P (2021). Corosolic acid inhibits colorectal cancer cells growth as a novel HER2/HER3 heterodimerization inhibitor. Br J Pharmacol.

[48] Qin Y, Yu Y, Yang C, Wang Z, Yang Y, Wang C (2021). Atractylenolide I inhibits NLRP3 inflammasome activation in Colitis-Associated Colorectal Cancer via suppressing Drp1-Mediated mitochondrial fission. Front Pharmacol.

[49] Zamorano-Leon JJ, Ballesteros S, de Las Heras N, Alvarez-Sala L, de la Serna-Soto M, Zekri-Nechar K (2019). Effect of pectin on the Expression of Proteins Associated with mitochondrial Biogenesis and Cell Senescence in HT29-Human colorectal adenocarcinoma cells. Prev Nutr Food Sci.

[50] Wang SQ, Cui SX, Qu XJ (2019). Metformin inhibited colitis and colitis-associated cancer (CAC) through protecting mitochondrial structures of colorectal epithelial cells in mice. Cancer Biol Ther.

[51] Wang Y, Sun X, Ji K, Du L, Xu C, He N (2018). Sirt3-mediated mitochondrial fission regulates the colorectal cancer stress response by modulating the Akt/PTEN signalling pathway. Biomed Pharmacother.

[52] Sun Y, Yang YM, Hu YY, Ouyang L, Sun ZH, Yin XF (2022). Inhibition of nuclear deacetylase Sirtuin-1 induces mitochondrial acetylation and calcium overload leading to cell death. Redox Biol.

[53] Yao W, Zhu S, Li P, Zhang S (2019). Large tumor suppressor kinase 2 overexpression attenuates 5-FU-resistance in colorectal cancer via activating the JNK-MIEF1-mitochondrial division pathway. Cancer Cell Int.

[54] Zhang Y, Wang M, Xu X, Liu Y, Xiao C (2019). Matrine promotes apoptosis in SW480 colorectal cancer cells via elevating MIEF1-related mitochondrial division in a manner dependent on LATS2-Hippo pathway. J Cell Physiol.

[55] Li H, He F, Zhao X, Zhang Y, Chu X, Hua C (2017). YAP inhibits the apoptosis and Migration of Human rectal Cancer cells via suppression of JNK-Drp1-Mitochondrial Fission-HtrA2/Omi Pathways. Cell Physiol Biochem.

[56] Kim YY, Yun SH, Yun J (2018). Downregulation of Drp1, a fission regulator, is associated with human lung and colon cancers. Acta Biochim Biophys Sin (Shanghai).

[57] Jieensinue S, Zhu H, Li G, Dong K, Liang M, Li Y (2018). Tanshinone IIA reduces SW837 colorectal cancer cell viability via the promotion of mitochondrial fission by activating JNK-Mff signaling pathways. BMC Cell Biol.

[58] Qian J, Fang D, Lu H, Cao Y, Zhang J, Ding R (2018). Tanshinone IIA promotes IL2-mediated SW480 colorectal cancer cell apoptosis by triggering INF2-related mitochondrial fission and activating the Mst1-Hippo pathway. Biomed Pharmacother.

[59] Zhuo F-F, Li L, Liu T-T, Liang X-M, Yang Z, Zheng Y-Z, et al. Lycorine promotes IDH1 acetylation to induce mitochondrial dynamics imbalance in colorectal cancer cells. Cancer Lett. 2023;573. 10.1016/j.canlet.2023.216364. 10.1016/j.canlet.2023.216364 37648148

[60] Zhang K, Zhang D, Wang J, Wang Y, Hu J, Zhou Y (2022). Aloe gel glucomannan induced colon cancer cell death via mitochondrial damage-driven PINK1/Parkin mitophagy pathway. Carbohydr Polym.

[61] Liskova V, Kajsik M, Chovancova B, Roller L, Krizanova O (2022). Camptothecin, triptolide, and apoptosis inducer kit have differential effects on mitochondria in colorectal carcinoma cells. FEBS Open Bio.

[62] He Y, Kan W, Li Y, Hao Y, Huang A, Gu H (2021). A potent and selective small molecule inhibitor of myoferlin attenuates colorectal cancer progression. Clin Transl Med.

[63] Wang SQ, Yang XY, Cui SX, Gao ZH, Qu XJ (2019). Heterozygous knockout insulin-like growth factor-1 receptor (IGF-1R) regulates mitochondrial functions and prevents colitis and colorectal cancer. Free Radic Biol Med.

[64] Liu W, Chen S, Xie W, Wang Q, Luo Q, Huang M (2023). MCCC2 is a novel mediator between mitochondria and telomere and functions as an oncogene in colorectal cancer. Cell Mol Biol Lett.

[65] Leo M, Muccillo L, Pranzini E, Barisciano G, Parri M, Lopatriello G, et al. Transcriptomic Analysis of Colorectal Cancer Cells Treated with oil production Waste products (OPWPs) reveals Enrichment of pathways of mitochondrial functionality. Cells. 2022;11(24). 10.3390/cells11243992. 10.3390/cells11243992 PMC9776412 36552757

[66] Kuznetsov AV, Javadov S, Margreiter R, Grimm M, Hagenbuchner J, Ausserlechner MJ (2021). Structural and functional remodeling of mitochondria as an adaptive response to energy deprivation. Biochim Biophys Acta Bioenerg.

[67] Zhang B, Liu Q, Wen W, Gao H, Wei W, Tang A (2022). The chromatin remodeler CHD6 promotes colorectal cancer development by regulating TMEM65-mediated mitochondrial dynamics via EGF and wnt signaling. Cell Discov.

[68] Cai WF, Zhang C, Wu YQ, Zhuang G, Ye Z, Zhang CS (2018). Glutaminase GLS1 senses glutamine availability in a non-enzymatic manner triggering mitochondrial fusion. Cell Res.

[69] Li L, Chen Q, Yu Y, Chen H, Lu M, Huang Y (2020). RKI-1447 suppresses colorectal carcinoma cell growth via disrupting cellular bioenergetics and mitochondrial dynamics. J Cell Physiol.

[70] Li J, Ye Y, Liu Z, Zhang G, Dai H, Li J (2022). Macrophage mitochondrial fission improves cancer cell phagocytosis induced by therapeutic antibodies and is impaired by glutamine competition. Nat Cancer.

[71] Wang Y, Subramanian M, Yurdagul A Jr., Barbosa-Lorenzi VC, Cai B, de Juan-Sanz J, et al. Mitochondrial fission promotes the continued clearance of apoptotic cells by macrophages. Cell. 2017;171(2):331–345e322. 10.1016/j.cell.2017.08.041. 10.1016/j.cell.2017.08.041 PMC5679712 28942921

[72] Mitochondrial fission in macrophages fuels phagocytosis of tumor cells (2022). Nat Cancer.

[73] Ding C, Shrestha R, Zhu X, Geller AE, Wu S, Woeste MR (2023). Inducing trained immunity in pro-metastatic macrophages to control tumor metastasis. Nat Immunol.

[74] Buck MD, O’Sullivan D, Geltink K, Curtis RI, Chang JD, Sanin CH (2016). Mitochondrial Dynamics Controls T Cell Fate through Metabolic Programming. Cell.

[75] He J, Shangguan X, Zhou W, Cao Y, Zheng Q, Tu J (2021). Glucose limitation activates AMPK coupled SENP1-Sirt3 signalling in mitochondria for T cell memory development. Nat Commun.

[76] Herkenne S, Ek O, Zamberlan M, Pellattiero A, Chergova M, Chivite I (2020). Developmental and Tumor Angiogenesis requires the Mitochondria-Shaping protein Opa1. Cell Metab.

[77] Barreto R, Mandili G, Witzmann FA, Novelli F, Zimmers TA, Bonetto A (2016). Cancer and Chemotherapy Contribute to muscle loss by activating Common Signaling pathways. Front Physiol.

[78] Zhong X, He X, Wang Y, Hu Z, Huang H, Zhao S (2022). Warburg effect in colorectal cancer: the emerging roles in tumor microenvironment and therapeutic implications. J Hematol Oncol.

