# Supplementary material for: Mitochondrial dynamics and colorectal cancer biology: mechanisms and potential targets
Source: Cell Commun Signal. 2024 Feb 1;22:91. doi: 10.1186/s12964-024-01490-4 (PMC10835948; doi:10.1186/s12964-024-01490-4)
Supplement: Supplementary file 1 — Additional file 1: Supplementary Table 1. The role of mitochondrial dynamics in CRC. [file 12964_2024_1490_MOESM1_ESM.docx]

***Supplementary table 1***. The role of mitochondrial dynamics in CRC.

| **Fission promotes cell proliferation and migration** | | | | |
| --- | --- | --- | --- | --- |
| **Models** | **Molecules/Compounds** | | **Findings** | **References** |
| CRC cell lines  CDX/PDX model  Model mice  human CRC tissues | **Molecules** | Drp1 | Mitochondrial fission factor Drp1 inhibits CRC cell apoptosis. | [31] |
|  |  | Drp1 | Activation of Drp1 promotes FAs oxidation, resulting in metabolic reprogramming in CRC cells and enhancement of the Wnt/β-catenin pathway. | [32] |
|  |  | *BRAF*^V600E^ | Mitochondrial fission reprograms glucose metabolism, promoting growth, invasion, and migration by activating the MEK/ERK signaling downstream of *BRAF*^V600E^ in CRC cells. | [33] |
|  |  | *RAS*^G12V^ | *RAS*^G12V^ promotes rapid mitochondrial fission, potentially through the induction of Drp1 due to an oncogenic MAPK signal. | [34] |
|  |  | ARF1-  IQGAP1 | ARF1 enhances the interaction between IQGAP1 with ERK and MEK, promoting mitochondrial fission and leading to colon tumorigenesis. | [35] |
|  |  | MTFR2 | Mitochondrial fission induces the proliferation, invasion and migration ability of CRC cells | [36] |
|  |  | miR-27a/*FOXJ3* | The miR-27a/FOXJ3 axis regulates mitochondrial dynamics and biogenesis, resulting in shorter, fewer, and punctate mitochondria. | [37] |
|  |  | OTUD6A | Overexpression of OTUD6A increases Drp1 levels, induces mitochondrial fragmentation, and enhances CRC cell proliferation. | [38] |
|  |  | Drp1 | Mitochondrial fission facilitates the development of doxorubicin-resistant colon cancer cells. | [39] |
|  |  | miR-17-5p | miR-17-5p induces mitochondrial fission, leading to reduced apoptosis and chemoresistance to 5-FU in CRC. | [40] |
|  |  | HMGB1/RAGE | ERK-mediated high Drp1 phosphorylation triggers autophagy via the RAGE/ERK pathway, resulting in chemoresistance. | [41] |
| CRC cell lines  CDX model  AOM/DSS model  human CRC tissues | **Compounds** | Ellagic acid | Ellagic acid inhibits CRC cell proliferation and induces widespread cell death by inhibiting Drp1-mediated mitochondrial fission. | [43] |
|  |  | Paris Saponin II | Paris Saponin II inhibits ERK1/2 phosphorylation and Drp1 via activating the NF-κB pathway, triggering cell apoptosis. | [44] |
|  |  | Sodium butyrate | Sodium butyrate induces cell cycle arrest at the G2/M phase and inhibits cell viability by suppressing mitochondrial fission. | [45] |
|  |  | ICG-001 | ICG-001 inhibits cell viability by inhibiting the phosphorylation of Drp1 at Ser616 and activating the ER stress response in CRC cells. | [46] |
|  |  | Corosolic acid | Corosolic acid exhibits anti-CRC activity by targeting the heterodimerization of HER2 and HER, inhibiting mitochondrial fission. | [47] |
|  |  | Atractylenolide I | Atractylenolide I suppresses NLRP3 inflammasome activation in the CAC by inhibiting mitochondrial fission, leading to apoptosis. | [48] |
|  |  | Pectin | Pectin inhibits mitochondrial fission and promotes senescence in CRC cells. | [49] |
|  |  | Mdivi-1 | Mdivi-1 inhibits mitochondrial fission, reduces the oxidative metabolism of CRC cells, and impairs cell proliferation. | [42] |
|  |  | Azelastine | Azelastine may inhibit ARF1-mediated mitochondrial fission via the ERK signaling pathway to suppress colon tumorigenesis. | [35] |
|  |  | Metformin | Metformin prevents H2O2-induced mitochondrial fission via activating the LKB1/AMPK pathway, thereby inhibiting CAC. | [50] |
| **Excessive fission induces apoptosis** | | | | |
| CRC cell lines  human CRC tissues | **Molecules** | SIRT3 | SIRT3-mediated fatal mitochondrial fission promotes CRC apoptosis by inhibiting the Akt/PTEN pathway. | [51] |
|  |  | SIRT1 | SIRT1 inhibition promotes mitochondrial acetylation and division, leading to mitochondrial calcium overload and CRC apoptosis. | [52] |
|  |  | LATS2 | Overexpression of LATS2 can overcome resistance to 5-FU by amplifying JNK-MIEF1-related mitochondrial fission. | [53] |
|  |  | Yap | Yap deficiency promotes excessive mitochondrial fission and HtrA2/Omi release through activation of the JNK/Drp1 pathway, ultimately inducing apoptosis. | [55] |
|  |  | Drp1 | The deficiency of Drp1 may be implicated in the progression of colon cancer. | [56] |
| CRC cell lines  PDX model | **Compounds** | Inauhzin | Inauhzin promotes mitochondrial acetylation and division, leading to mitochondrial calcium overload and apoptosis. | [52] |
|  |  | Matrine | Matrine activates MIEF1-related mitochondrial fission and inhibits SW480 cells survival via the LATS2-Hippo pathway. | [54] |
|  |  | Tanshinone IIA | Tanshinone IIA induces apoptosis and inhibit CRC cell proliferation through the promotion of mitochondrial fission by activating JNK-Mff and Mst1-Hippo signaling. | [57,58] |
|  |  | Lycorine | Lycorine promotes SIRT1-dependent acetylation of mitochondrial proteins, which significantly exacerbates oxidative stress and mitochondrial fission in CRC. | [59] |
|  |  | *Aloe gel* glucomannan | *Aloe gel* glucomannan upregulates mitophagy and mitochondrial fission signaling in CT26 cells, , ultimately driving cancer cell death. | [60] |
|  |  | Camptothecin,  Triptolide,  AIK | Camptothecin, Triptolide, and AIK target mitochondria through the process of fission and affect the induction of apoptosis in CRC cells. | [61] |
|  |  | YQ456 | YQ456 could dephosphorylate Drp1 at S637 in CRC cells, resulting in sustained mitochondrial fission and cell death. | [62] |
| **Fusion attenuates oxidative stress-induced mitochondrial damage** | | | | |
| CRC cell lines  AOM/DSS model  human CRC tissues | **Molecules** | IGF1R | Heterozygous knockdown of IGF-1R protects colonic epithelial cells from oxidative stress and prevents colon tumorigenesis by activating mitochondrial fusion function. | [63] |
|  |  | *MCCC2* | *MCCC2* knockdown induces mitochondrial fusion, thereby inhibiting CRC cell proliferation, invasion, and migration. | [64] |
|  |  | OPWPs,  Hydroxytyrosol | OPWPs extracts and hydroxytyrosol promote mitochondrial fusion via PPARγ/PGC-1α axis, causing proliferation inhibition and apoptosis in CRC cells. | [65] |
| CRC cell lines | **Compounds** | 2-DG | The treatment with 2-DG treatment induces the activation of AMPK pathway, leading to increased mitochondrial fusion and decreased fission, ultimately inhibiting glycolysis in cancer cells. | [66] |
| **Excessive fusion promotes cell proliferation and migration** | | | | |
| CRC cell lines  CDX/PDX model  AOM/DSS model  human CRC tissues | **Molecules** | *CHD6*-TMEM65 | *CHD6* induces TMEM65-mitochondrial fusion, leading to increased ATP production and promoting CRC progression through the activation of EGF and Wnt/β-catenin signaling. | [67] |
| CRC cell lines  CDX model | **Compounds** | RKI-1447 | Treatment with RKI-1447 inhibits mitochondrial fusion in CRC cells, thereby promoting apoptosis related to ER stress. | [69] |

Note: AIK, apoptosis inducer kit; AOM/DSS, Azoxymethane/Dextran Sodium Sulfate; CAC, colitis-associated cancer; CDX, cell derived xenograft; CRC, colorectal cancer; Drp1, dynamin-related protein 1; ER, endoplasmic reticulum; ETC, electron transport chain; FAs, fatty acids; Mff, mitochondrial fission factor; Mfn, mitofusin; MMP, mitochondrial membrane potential; mPTP, mitochondrial permeability transition pore; OPA1, optic atrophy 1; OPWPs, oil production waste products; OXPHOS, oxidative phosphorylation; PDX, patient-derived xenograft; ROS, reactive oxygen species; TCA, tricarboxylic acid cycle; 5-FU, 5-Fluorouracil.
